# Risk factors for venous thromboembolism following fractures isolated to the foot and ankle fracture

**DOI:** 10.1371/journal.pone.0276548

**Published:** 2022-10-20

**Authors:** Michael J. Gouzoulis, Peter Y. Joo, Alexander J. Kammien, William M. McLaughlin, Brad Yoo, Jonathan N. Grauer

**Affiliations:** Department of Orthopaedics and Rehabilitation, Yale School of Medicine, New Haven, CT, United States of America; Ningbo University, CHINA

## Abstract

**Objective:**

Venous thromboembolism (VTE) is an uncommon, but potentially morbid, complication following foot and ankle fractures. Current standard is to not administer thromboprophylaxis to patients with such injuries. Nonetheless, patient and fracture factors might affect this risk/benefit consideration. The goal of this study was to determine what patients are most at risk.

**Methods:**

The M53Ortho Pearldiver database was used to identify patients with fractures isolated to the foot and ankle that were treated non-operatively or operatively. Patients with pilon, other appendicular fractures remote from the foot and ankle, and other traumatic injuries were excluded. The 90-day occurrence of VTE was identified based on codes for deep vein thrombosis or pulmonary embolism. Characteristics of those patients who did and did not have VTEs were compared using chi-square analyses. Multivariate logistical regression was then performed to determined factors independently associated with VTE. Finally, timing of VTE relative to fracture was analyzed.

**Results:**

A total of 298,886 patients with isolated foot or ankle fractures were identified, of which 1,661 (0.56%) had VTE in the 90 days following fracture. In terms of timing, 27.3% occurred in the first week, and 49.8% occurred in the first three weeks. Independent risk factors for VTE included (in decreasing order):prior VTE (odd ratio [OR] = 25.44), factor V Leiden (OR = 24.34), active cancer (OR = 1.84), specific fracture relative to metatarsal fracture (multiple fractures [OR: 1.51], ankle fracture [OR = 1.51], and calcaneus fracture [OR = 1.24]), surgical treatment (OR = 1.41), male sex (OR = 1.19), greater Elixhauser index (OR = 1.05), and increasing age (OR:1.05 per decade) (p<0.05 for each).

**Conclusions:**

The present study found that, although only 0.56% of isolated foot and ankle fractures had a VTE within ninety days. Defined risk factors, such as Factor V Leiden, prior VTE, surgical treatment, active cancer, specific fracture patterns, and surgical treatment significantly affected the odds of their occurrence.

## Introduction

Orthopedic injuries and surgery are well-known to predispose patients to venous thromboembolism (VTE) [1–8], defined as the occurrence of deep vein thrombosis (DVT) and/or pulmonary embolus (PE). VTE thromboprophylaxis may be recommended after injury and surgery if the benefits are thought to outweigh the risks [9, 10]. Nonetheless, the risk/benefit ratio of VTE thromboprophylaxis makes this not typically recommended for isolated foot and ankle fractures.

VTE thromboprophylaxis is commonly given following many orthopaedic procedures at or below the hip due to the high risk associated with the surgery. Hip fracture surgery has a risk of VTE following surgery reported to be 2.5%-6.6% [11–13]. Total hip and total knee arthroplasty have a risk of VTE following surgery reported to respectively be 0.1%-4.2% [14–18] and 1.0%– 2.6% [16–19]. Based on such findings, several national organizations, such as American Academy of Orthopaedic Surgeons and American College of Chest Physicians, recommend the use venous thromboembolism thromboprophylaxis after these procedures [20].

Following foot and ankle surgery, Huntley et al. examined 23,212 patients in National Surgical Quality Improvement Program (NSQIP) database and found an incidence of VTE of 0.6% [21]. Basques et al. looked at 4,412 patients in NSQIP undergoing surgery for ankle fracture and found the incidence of VTE to be 0.8% [22]. Shibuya et al. examined 75,664 patients in National Trauma Data Bank (a database focused on higher energy trauma patient) undergoing foot and ankle fracture surgery and found the incidence of DVT to be 0.28% and the incidence of PE risk to be 0.21% [23]. Ahmad et al looked at 2774 patients in Misys Vision and found an incidence of VTE of 0.79% after foot and ankle surgery.[24] Although VTE risk was relatively low in all these described studies, they suggested that factors such as age could increase the risk of VTE in the foot and ankle fracture population [21, 22].

Based on the reported low incidence of VTE following foot and ankle fractures, the American Orthopaedic Foot and Ankle Society (AOFAS) does not currently recommend VTE thromboprophylaxis following foot and ankle surgery [25]. The American College of Chest Surgeons’ guidelines similarly do not recommend VTE thromboprophylaxis for injuries below the knee, however they note that this group of patients are heterogenous, and that some patients, such as those with a prior VTE, might benefit [25]. Notably, patient specific risk factors are not delineated in these considerations due to lack of published evidence [25].

Despite the low incidence of VTE following foot ankle fractures, it is hypothesized that specific patients likely have a significant increase risk, such as those with surgical management or clotting risk factors. Noting that not all patients with varied foot and ankle fractures are at the same risk of VTE, the current study used a large national insurance claims database to retrospectively analyze patient, fracture, and management variables to identify timing and predictive factors for VTE in patients with isolated foot and ankle fractures.

## Methods

### Patient sample

Data for the present study were obtained through a retrospective review of the 2015 to 2020 M53 Ortho Pearldiver database. The Pearldiver database is a large national claims database that contains patient information on over 53 million patients in both the inpatient and outpatient setting in the United States. It contains information all patients with both commercial and government (Medicare, Medicaid) insurance, and identifies patients based on billing. The Yale IRB determined that the investigator is not engaged in research involving human subjects. As such, IRB review and approval are not required.

Patients with foot and ankle fractures were identified using International Classification of Disease tenth edition (ICD10) codes to identify fractures of the ankle, talus, calcaneus, tarsal, metatarsals bones, or multiple of these. ICD10 codes used can be found in S1 Fig. Patients were excluded if they were less than eighteen years of age, had pilon fracture, or had concurrent non-foot ankle fractures or trauma. Further, patients were excluded if they did not remain active in the dataset for at least 90 days.

### Patient and fracture characteristics

Descriptive characteristics abstracted from the database included age and sex. Comorbidity status was approximated with the Elixhauser Comorbidity Index (ECI), an established index that provides a score to determine the overall comorbidity burden of patients. ECI is a validated comorbidity index that has been shown to similar to or superior to other indexes for determining adverse event following orthopaedic procedures [26–28]. Additionally abstracted was a specific history of clotting risk factors (Factor V Leiden, prior VTE, active cancer, prior myocardial infarction). Patients who underwent surgery were identified using the Current Procedural Terminology (CPT) codes.

The occurrence and timing of VTE in the 90-days post injury were then identified based on the occurrence of deep DVE and/or PE. 90-days post injury was chosen to adequately compared the non-surgical and surgical patients. Additionally, a prior study has shown that when comparing thirty-day versus ninety-day, some complications are missed when only following the patients for thirty [29]. The timing of the VTE defined as number of days following the initial fracture.

### Statistical analysis

Univariate comparisons were made using chi-square tests to determine the difference between those who did and did not experience VTE. Univariate analysis compared age, sex, ECI, clotting risk factors, type of fracture(s), and occurrence of surgery between the two groups.

Multivariable logistical regression was then performed to control for the confounding effect that different independent variables might have on incidence of VTE. This included the following independent variables: age, sex, ECI, clotting risk factors, type of fracture(s), active cancer, prior myocardial infarction (MI), and occurrence of surgery.

Univariate analyses and logistical regression were performed using Pearldiver statistical software (Pearldiver Inc, Colorado Spring, CO). Figures were created using GraphPad Prism 9 (GraphPad Software, San Diego, CA).

## Results

### Study population

**Fig 1:**
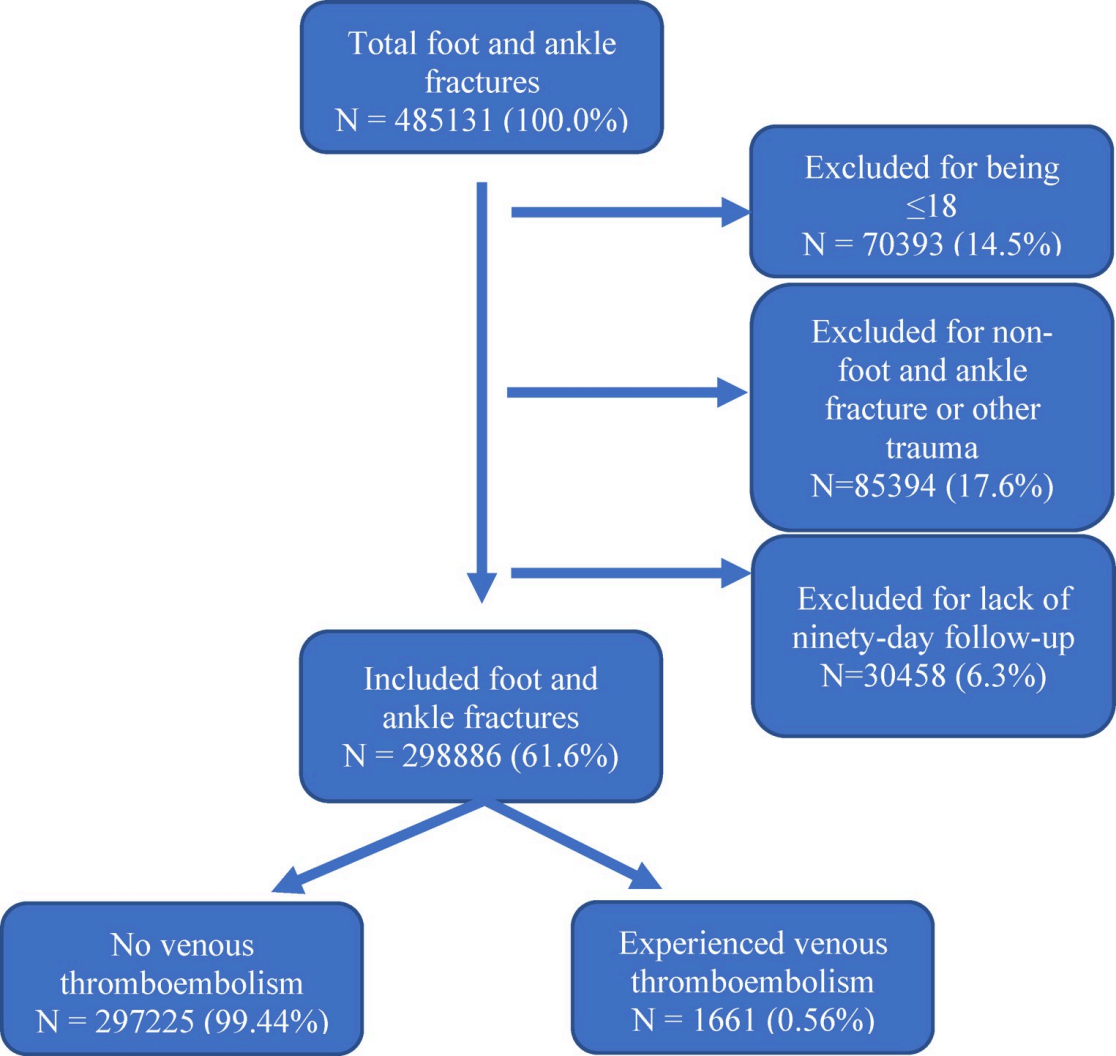
Included patient sample with exclusion criteria. Algorithm flowchart for initial patient inclusion, along with all exclusions applied to the sample. Total patients included and excluded are shown.

A total of 485,131 patients with any foot or ankle fractures were identified. Patients were excluded if they were eighteen years of age or under (14.5%), had pilon fracture or other concurrent non-foot and ankle fractures (17.6%), or if they did not have ninety days of follow-up (6.3%). After these exclusions, there were 298,886 (61.6%) patients included in the study (Fig 1).

**Fig 2:**
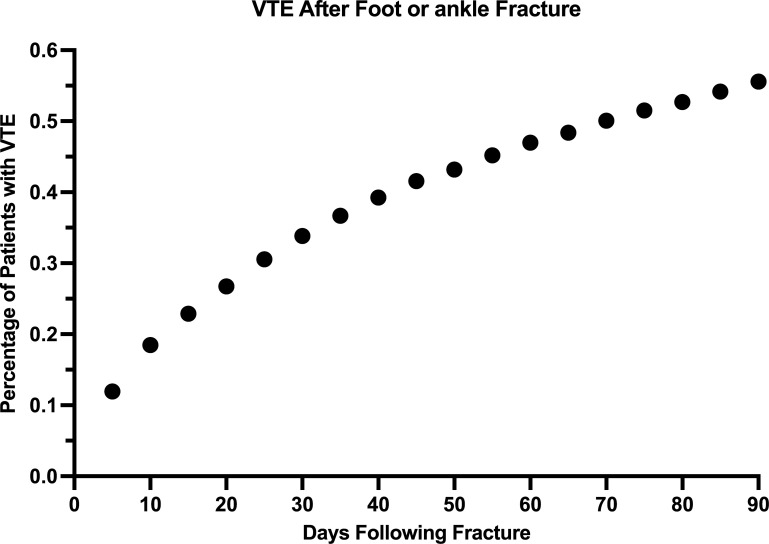
Incidence of venous thromboembolism following fracture or surgery. Timing of Venous Thromboembolism following a foot and ankle fracture. Nearly half of venous thromboembolism (49.8%) occurred within the first three weeks.

Of the study population, VTE was noted within 90 days of fracture for 1661 (0.56%). From a timing perspective, in the first week there were 454 VTEs (27.3% of 90-day VTEs) and in the first three weeks there were 827 VTEs (49.8% of 90-day VTEs). Fig 2 shows the temporal distribution of when patients had a VTE 90 days following fracture.

Patient characteristics of all identified isolated foot an ankle fractures, those without VTE, and those with VTE are shown in Table 1. For all patients identified for the study, the average age was 54.5 (standard deviation [SD]: 16.5), the average ECI was 4.4 (SD: 3.8), nonsurgical management was done for 285,180 (95.4%), surgical management was done for 13,706 (4.6%). The most common type of fracture of all patients identified for the study was a metatarsal fracture (52.3%), followed by ankle fractures (31.8%).

**Table 1:** Patient characteristics and related variables of those without and with VTE following isolated foot or ankle fractures.

	All Isolated Foot and Ankle Fractures	No VTE	VTE	P—value
Sample Size	298,886	297,225	1661	
Age	54.5 (16.5)	54.5 (16.5)	59.7 (14.9)	**< 0.001**
Sex				**0.01**
Female	212,923 (71.2%)	211,786 (71.2%)	1,137 (68.4%)	
Male	85,963 (28.8%)	85,439 (28.8%)	524 (31.6%)	
ECI:				**< 0.001**
Mean (SD)	4.4 (3.8)	4.4 (3.7)	7.3 (4.8)	
Median (IQR)	3 (2–5)	3 (2–5)	7 (4–9)	
Clotting Risk Factors				
Factor V Leiden	857 (0.3%)	797 (0.3%)	60 (3.6%)	**< 0.001**
Prior VTE	4,116 (1.4%)	3,982 (1.3%)	224 (13.5%)	**< 0.001**
Active Cancer	12,790 (4.3%)	12,611 (4.2%)	179 (10.8%)	**< 0.001**
Prior MI	16,061 (5.4%)	15,873 (5.3%)	188 (11.3%)	**< 0.001**
Fracture Types				
Ankle	89,914 (30.1%)	89,251 (30.0%)	663 (39.9%)	**< 0.001**
Talus	12,059 (4.3%)	12,002 (4.4%)	57 (3.4%)	0.23
Calcaneus	19,216 (6.4%)	19,112 (6.4%)	104 (6.3%)	0.82
Tarsal	16,437 (5.5%)	16,377 (5.5%)	60 (3.6%)	**< 0.001**
Metatarsal	147,155 (49.2%)	146,488 (49.3%)	667 (40.2%)	**< 0.001**
Multiple F&A fractures	14,485 (4.9%)	14,372 (4.8%)	113 (6.8%)	**< 0.001**
Treatment Type				**< 0.001**
Nonsurgical	285,180 (95.4%)	283,634 (95.4%)	1,546 (93.1%)	
Surgical	13,706 (4.6%)	13,591 (4.6%)	115 (6.9%)	

**Bolding** indicates significance, p < 0.05

VTE = Venous Thromboembolism

ECI = Elixhauser Comorbidity Index

F&A = Foot and Ankle

### Factors associated with VTE

By univariate analysis, those with VTE were more likely to be older (54.5 vs 59.7, p<0.001), male (28.8% vs 31.6%, p = 0.01), and have a higher ECI (4.4 vs 7.3, p<0.001). Those with VTE were also more likely to have Factor V Leiden (3.6% vs 0.3%), prior VTE (13.5% vs 1.3%), active cancer (4.2% vs 10.8%, p<0.001) and have had surgery for their fracture (6.9% vs 4.6%) (p<0.001 for reach). Patients who had a VTE were more likely to have an ankle fracture (39.9% vs 30.0%) and multiple fractures (6.8% vs 4.8%), while being less likely to have a tarsal fracture (3.6 vs 5.5%) or metatarsal fracture (40.0% vs 49.3%). These results are shown in Table 1.

By multivariate analysis, factors associated with VTE (in decreasing order) were: prior VTE (OR: 25.44, 95% CI: 19.70–33.29), factor V Leiden (OR = 24.34, 95% CI: 16.96–33.29), active cancer (OR = 1.84, 95% CI: 1.30–2.60), specific fracture relative to metatarsal fracture (multiple fractures [OR: 1.51, 95% CI: 1.22–1.85], ankle fracture [OR = 1.51, 95% CI: 1.35–1.69], and calcaneus fracture [OR = 1.24, 95% CI: 1.00–1.53]), surgical treatment (OR = 1.41, 95% CI: 1.15–1.72), male sex (OR = 1.19), greater Elixhauser index (OR = 1.05, 95% CI: 1.04–1.06), and increasing age (OR:1.05 per decade, 95% CI: 1.01–1.08) (p<0.05 for each).These results are shown in Table 2.

**Table 2:** Multivariate analyses of risk factors for VTEs after foot or ankle fractures.

	Odds Ratio	95% Confidence Interval	P–Value
Age (Per Decade)	1.05	1.01–1.08	**0.001**
Sex			
Female	REF	REF	REF
Male	1.19	1.07–1.32	**0.002**
ECI	1.05	1.04–1.06	**0.001**
Clotting Risk Factors			
No Risk Factors	REF	REF	REF
Prior VTE	25.44	19.70–33.29	**< 0.001**
Factor V Leiden	24.34	16.96–33.29	**< 0.001**
Active Cancer	1.84	1.30–2.60	**< 0.001**
Prior MI	1.03	0.71–1.50	0.87
Fracture Type			
Ankle	1.51	1.35–1.69	**< 0.001**
Talus	1.07	0.80–1.40	0.63
Calcaneus	1.24	1.00–1.53	**0.048**
Tarsal	0.2	0.69–1.21	0.58
Metatarsal	REF	REF	REF
Multiple F&A Fractures	1.51	1.22–1.85	**< 0.001**
Treatment type			
Nonsurgical	REF	REF	REF
Surgical	1.41	1.15–1.72	**< 0.001**

Controlled for age, sex, ECI, clotting risk factors, surgical treatment, type of fracture/multiple fractures.

Bolding indicates of p < 0.05.

REF = referent variable

VTE = Venous Thromboembolism

ECI = Elixhauser Comorbidity Index

MI = Myocardial Infarction

## Discussion

VTE is a rare event after isolated foot and ankle fractures, and current guidelines do not recommend routine VTE prophylaxis following related fractures/surgeries. Prevention strategies such as prophylactic anticoagulation is based around identification of patient underlying risk factors, both acquired and inherited, that place one at an increased risk of VTE [22, 30–32]. There remains a paucity of evidence on which patients, and specifically which risk factors, would benefit most from postoperative VTE prophylaxis. To our knowledge, the present study is the first to evaluate specific surgical and non-surgical risk factors for VTE after isolated foot and ankle fractures in 298,886 individuals using PearlDiver, a large national insurance claims database.

The current study found an overall VTE incidence of 0.56% for all foot and ankle fractures, which is like past studies [21–23, 33, 34]. Basques et al reported a 0.8% incidence of VTE within thirty days following ankle surgery using the NSQIP database [22]. Jameson et al. reported a lower incidence of VTE of 0.285% within ninety days following foot and ankle surgery using the Hospital Episode Statistics from NHS [33]. Patients with foot and ankle fractures represent a very heterogenous population, with it being a common injury amongst polytrauma patients [35], while also being an injury common in all ages, from pediatrics to elderly [36, 37]. To focus more on foot and ankle fractures, patients with any other fractures or injuries were excluded, and the study focused exclusively on the adult population. The reasoning for this was to create a homogenous study population to best focus on the effect of the fracture on the patient’s risk of VTE. Multivariate analysis was then performed as univariate analyses can be misleading if patient injury fractures overlap. Some of the ORs produced from multivariate analyses are quite large and bear further discussion.

The strongest association with VTE was found to be a history of prior VTE. Patients with a prior history of VTEs were roughly twenty-five times as likely to have a VTE following a fracture. Personal history is commonly cited as a significant risk factor for recurrent VTEs, and the Caprini criteria labels it as “high risk” and recommends thromboprophylaxis to these patients [38, 39].

The next greatest association with VTE was found to be the factor V Leiden. Patients with Factor V Leiden were nearly twenty-five times increased odds of VTE. Factor V Leiden is commonly cited as greatly increasing risk of venous thromboembolisms and is one of the genetic predispositions that play a role in half of idiopathic VTEs [40], and has been found to increase VTE risk overall in many orthopaedic indications [41].

The presence of active cancer was associated with a nearly two-fold increased odds of VTE. Active cancer has been thoroughly investigated as a risk factor for VTE [42, 43]. The mechanism of cancer leading to VTE is thought to be due to cancer causing hypercoagulability, potentially as a result of procoagulant factor being released [42]. To add to that, active cancer has been shown to be a significant risk factor following many orthopaedic procedures[44, 45]. However, some literature has suggested that thromboprophylaxis does not lead to significantly different rates of VTEs in these patients [45].

Surgical treatment was associated with VTE. Major surgery—in particular orthopedic surgery- is a well-known risk factor for VTE. In the present study, surgery alone was found to increase odds of VTE by 41% compared to no surgery. However, evidence on the prevention of symptomatic VTE with the use of chemical prophylaxis after foot and ankle surgery remains inconsistent and controversial. The American Orthopaedic Foot and Ankle Society in their position statement on the use of VTE prophylaxis after foot and ankle surgery from 2020 concluded that there is currently insufficient data on attributed risks of VTE after foot and ankle surgery to recommend for or against routine prophylaxis [25]. The United Kingdom’s National Institute of Clinical Excellence (NICE) guideline on VTE prophylaxis after foot and ankle surgery from 2018 recommends chemical prophylaxis when patients require lower limb immobilization up to 42 days, total anesthesia time is greater than 90 minutes, or when the risks of VTE outweigh risks of bleeding [32]. Immobilization and non-weightbearing status have been shown to be known risk factors that increases the risk of VTE after foot and ankle surgery [46]. We found that patients are at the highest risk for VTE within the first 3 weeks which correlates with incisional healing time.

Finally greater comorbidity burden and age were associated with VTE. For every point increase in ECI, there was a respective 5% increase OR, and for every decade of life, there was a respective increase of 5% VTE incidence. Comorbidity burden has been demonstrated in other orthopaedic indications to significantly increase severe adverse events, including venous thromboembolism [47, 48]. Increasing age, especially focused on the elderly population, has been studied extensively in orthopaedics surgeries, and is often a driving force for many complications, including VTE [49, 50]. Although the overall percentage increase for comorbidity burden and age seems small, due to the scaling nature of the OR, these factors can play a significant role in a patient’s risk.

There were several limitations to the current study. As with any retrospective database studies, the study is dependent on the administrative coding reported. Additionally, the study is reliant on that patients with VTE were properly screened and image in order to be diagnosed. Although standard of care currently does not recommend prophylactic anticoagulation for patients with foot and ankle fractures, it can’t be determined if select patients were prescribed prophylaxis. It is also not possible to know if any patients died during this period. It can also not be determined what indication there was for the initial Factor V Leiden diagnosis in patients, which could contribute to the strong correlation. Lastly, although exclusion criteria were applied, and multiple risk factors for VTE tracked, it is possible that other variables could have been present and skewed study findings.

Although current foot and ankle fracture guidelines do not recommend thromboprophylaxis for such injuries [25], there is the recommendation patients should be stratified and have prevention plans tailored to their individual risk level. The present study identifies and quantifies risk factors for VTE and finds that almost half of these occur in the three weeks. Notably, conditions such as Factor V Leiden and prior history of VTE, drastically increases one risk of subsequent VTE following a fracture. While we do not describe a risk assessment methodology, this study highlights a current gap in existing literature and guidelines on which foot and ankle fracture patients may benefit most from VTE prophylaxis.

## Supporting information

S1 Fig ICD10 codes for fractures and VTE. Overview of ICD10 coding for fractures, deep vein thrombosis, and pulmonary embolism.
(ZIP)
