# Supplementary figures and images for: Risk factors for venous thromboembolism following fractures isolated to the foot and ankle fracture
Source: PLoS One. 2022 Oct 20;17(10):e0276548. doi: 10.1371/journal.pone.0276548 (PMC9584400; doi:10.1371/journal.pone.0276548)

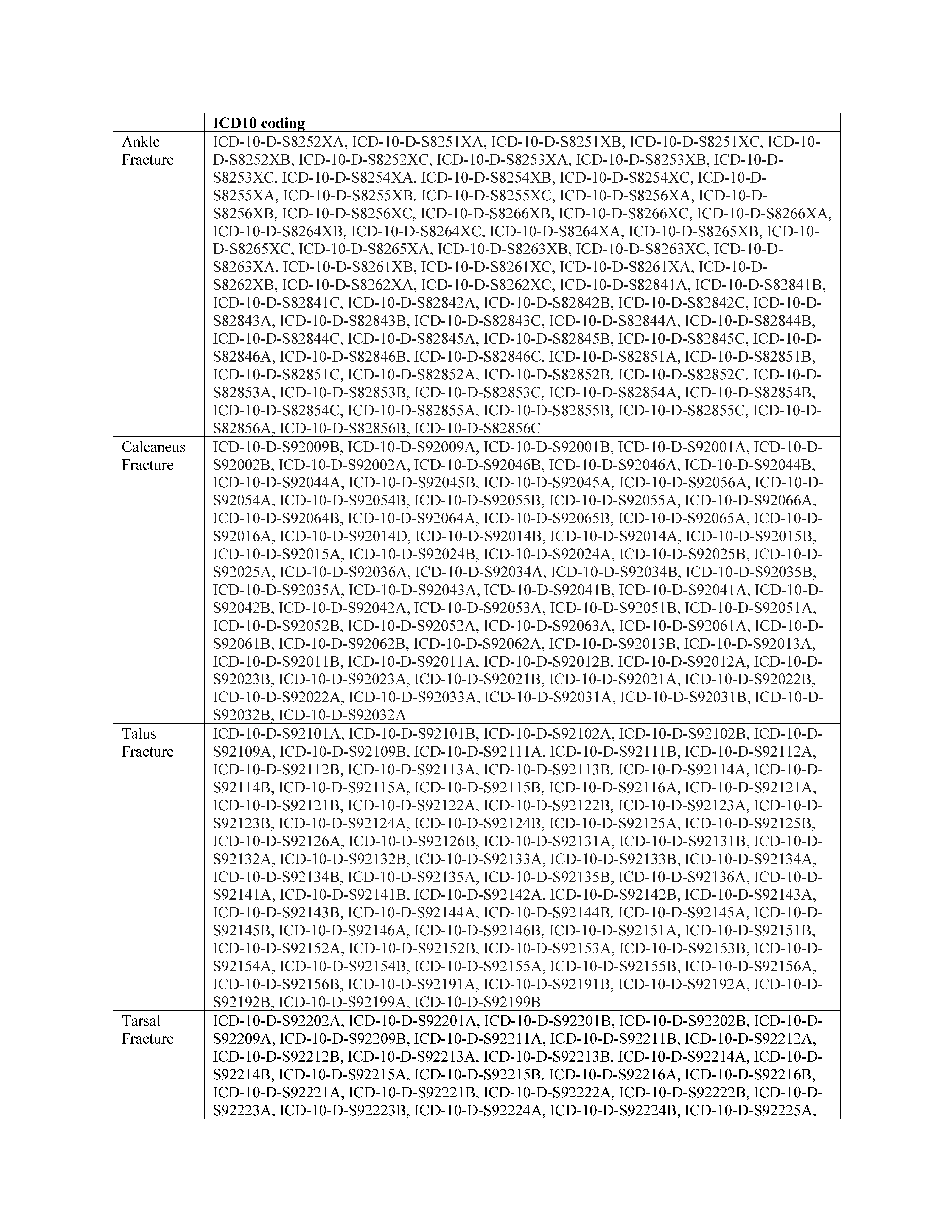

Supplement: S1 Fig — Overview of ICD10 coding for fractures, deep vein thrombosis, and pulmonary embolism. (ZIP) [file pone.0276548.s001.zip › S1 Fig_Page_1.tiff]
